# Self-assembled π-conjugated chromophores: preparation of one- and two-dimensional nanostructures and their use in photocatalysis

**DOI:** 10.1039/d4nr00383g

**Published:** 2024-04-04

**Authors:** David Cappelletti, Marianna Barbieri, Alessandro Aliprandi, Michele Maggini, Luka Đorđević

**Affiliations:** a Department of Chemical Sciences, University of Padova via Marzolo 1 35131 Padova Italy luka.dordevic@unipd.it

## Abstract

Photocatalytic systems have attracted research interest as a clean approach to generate energy from abundant sunlight. In this context, developing efficient and robust photocatalytic structures is crucial. Recently, self-assembled organic chromophores have entered the stage as alternatives to both molecular systems and (in)organic semiconductors. Nanostructures made of self-assembled π-conjugated dyes offer, on the one hand, molecular customizability to tune their optoelectronic properties and activities and on the other hand, provide benefits from heterogeneous catalysis that include ease of separation, recyclability and improved photophysical properties. In this contribution, we present recent achievements in constructing supramolecular photocatalytic systems made of chromophores for applications in water splitting, H_2_O_2_ evolution, CO_2_ reduction, or environmental remediation. We discuss strategies that can be used to prepare ordered photocatalytic systems with an emphasis on the effect of packing between the dyes and the resulting photocatalytic activity. We further showcase supramolecular strategies that allow interfacing the organic nanostructures with co-catalysts, molecules, polymers, and (in)organic materials. The principles discussed here are the foundation for the utilization of these self-assembled materials in photocatalysis.

## Abstract 2

Nanostructures obtained from self-assembled organic dyes are emerging as alternatives to molecular and semiconductor photocatalysts. With their unique properties, they offer a promising way to bridge homo- and heterogeneous (photo)catalysis.

## Graphical abstract

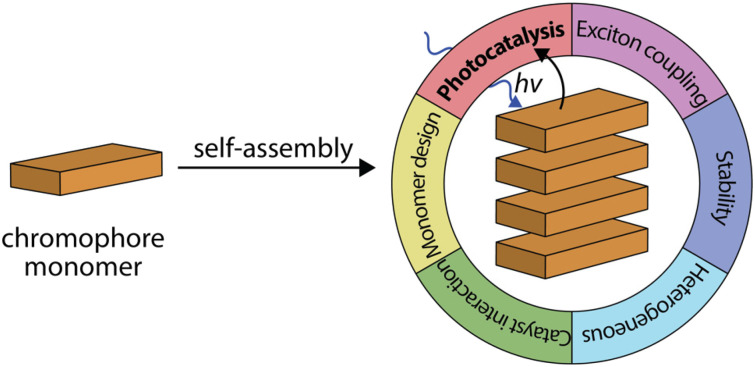

## Introduction

Due to increasing global energy demands, in addition to limited fossil fuels and environmental concerns, the conversion of solar light into energy or chemicals is gaining prominence. A constant source of inspiration for developing light-harvesting systems is natural photosynthesis, in which a preorganized assembly of chromophores performs conversion of light into chemical energy.^1–8^ While natural light-harvesting structures are usually organized *via* complex protein environments, some systems lack the support of a protein matrix. For example, in green sulphur photosynthetic bacteria, the so-called chlorosomes are based solely on pigments made of self-assembled chromophores.^9^ In these organisms, the energy absorbed by molecular antennas is ultimately used to transform substrates, such as carbon dioxide and hydrogen sulphide, into organic compounds, water and sulphur. While achieving such complex transformations in artificial systems is still far from our reach, the ability to prepare photoactive assemblies simply through self-assembly of chromophores has fascinated scientists and brought forward several exceptional systems in the last few years.

This contribution provides an overview of the emerging field of self-assembled organic chromophores and their application in photocatalysis. Supramolecular polymers composed of chromophores are arrays formed by monomeric dye units interconnected through directional secondary interactions, such as metal–ligand coordination, π–π stacking, hydrogen bonding, or a combination of these. It is noteworthy that the first observation of supramolecular dye polymers dates back to the late 1930s, when Scheibe^10^ and Jelley^11^ reported studies on cyanine aggregates and their photophysical properties. Other seminal contributions date back to the late 1980s, when Aida and co-workers reported on an amphiphilic porphyrin that formed co-facial stacks in aqueous media.^12^ Since these pioneering works and with the advent of supramolecular polymers,^13^ the field has been constantly evolving.^14–25^ Supramolecular polymers are also finding applications in energy-related applications^26^ with recent literature reviewing the photocatalytic application of perylene diimides (PDIs)^27^ and other systems for H_2_ production and CO_2_ reduction.^28^

**Fig. 1:**
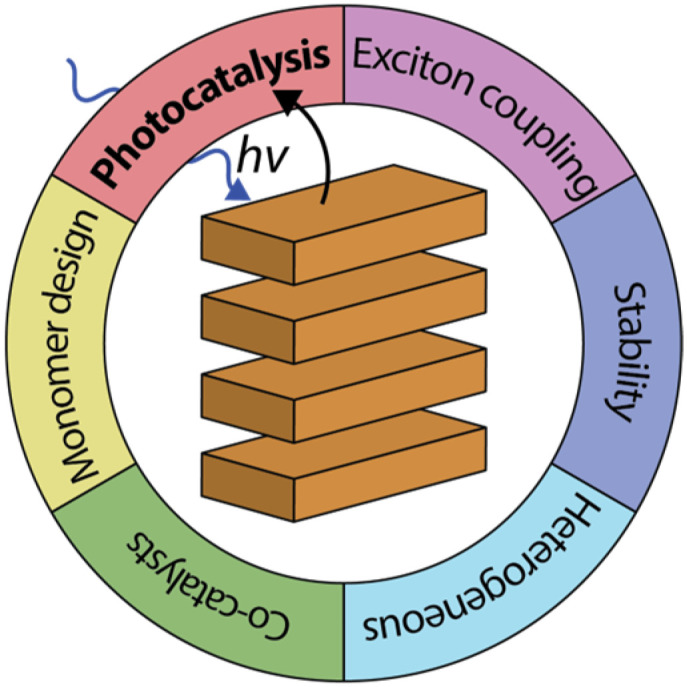
Organic dye aggregates used in photocatalysis have several advantages compared to their monomeric or semiconductor counterparts.

Here, we will focus on organic dyes that self-assemble through π–π stacking, as they can form robust and ordered aggregates in water. We organise our discussion by strategies that can be used to prepare ordered aggregates with photocatalytic applications. In these aggregates, the constituent monomeric chromophores can undergo electronic coupling, leading to emerging properties that enable solar energy conversion (Fig. 1). The spotlight of this work is on one- or two-dimensional nanostructures obtained through dye self-assembly in water, aqueous solutions containing salts, or solvent mixtures containing water. The ability to perform modern chemical reactions in water, paired with effective recycling strategies for catalysts, represents a cornerstone for sustainable and green chemistry. Additionally, water is a unique medium for self-assembly since biological systems excel at controlling supramolecular polymerization in aqueous media. In this context, synthetic self-assembled chromophores can combine the bioinspired aspect from natural systems with the synthetic accessibility and tunability of artificial molecules.

## Self-assembled chromophores in photocatalysis

**Fig. 2:**
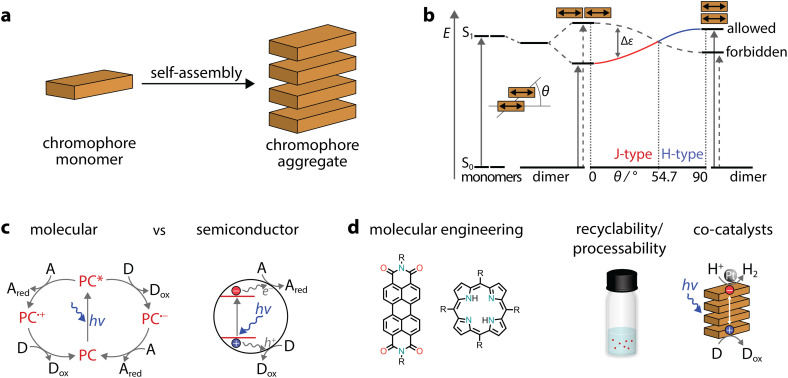
Overview of self-assembled chromophores and their use in photocatalysis. (a) Schematic representation of the self-assembly of a chromophore into an aggregate. (b) Schematic energy diagram for the exciton coupling of dyes with transition dipole moments and allowed transitions in J-type and H-type dimers. (c) Schematic representation of molecular *versus* semiconductor photocatalytic processes. (d) Self-assembled dye nanostructures combine advantages of homogeneous molecular photosensitizers (molecular engineering of monomers) and heterogeneous catalysts (stability, recyclability, and processability) for sacrificial photocatalysis.

When dyes self-assemble through π–π stacking (Fig. 2a), their photophysical properties can be drastically affected. In comparison to the monomer, aggregates can either exhibit blue-shifted (hypochromic shift) or red-shifted (bathochromic shift) absorption bands and are commonly called H- or J-aggregates, respectively. These changes can be explained through Kasha's exciton coupling theory,^29^ which is summarized in Fig. 2b. Taking a co-facially stacked dimer as an example, the transition dipole moments (TDM) of the monomers are oriented parallel to each other (slip angle of *θ* = 90°, H-aggregate, right side). In the other borderline case, the transition dipole moments are in line (*θ* = 0°, J-aggregates, left side). In addition, there are several deviations from the two ideal situations, but these are discussed elsewhere.^30^ While we exemplified the case of a dimer, the discussion on transition dipole moments and the transition energy also applies to large aggregates. Therefore, in aggregates the question of which chromophore is excited becomes irrelevant, as the excitation is rather delocalized and should be treated as a superposition of locally excited states.

In molecular photoredox catalysis,^31^ the photocatalyst is promoted to the excited state, which can undergo two possible pathways (Fig. 2c). Through oxidative or reductive quenching mechanisms, radicals are formed and are more reactive than the neutral precursors. Unlike in molecular photocatalysts, in semiconductors, excitation through incident photons leads to the generation of electron–hole pairs (excitons), followed by their separation into free charges, which ultimately drive redox reactions (Fig. 2c).^32^ Depending on the distance between the electron–hole pairs, excitons can be either localized on the same monomer (Frenkel exciton) or diffused over several monomers (charge transfer excitons). Indeed, the formation of π–π stacks could allow enhanced separation and migration of the photogenerated carriers, minimizing recombination which is highly desirable in photocatalysis.^25^ For example, crystalline nanostructures, obtained through interchain π–π stacking of polymeric fluorenes,^33,34^ were found to exhibit long-range exciton transport that is beneficial for light-driven H_2_ evolution.^35^ Thus, highly ordered organic aggregates could have advantages in photocatalytic processes.

Using self-assembled dye polymers could offer additional benefits in photocatalysis (Fig. 2d). First, molecular engineering and customizability can be achieved through a multitude of organic chemistry procedures, with changes in the chemical structure then being translated to an aggregate. Second, further chemical modifications can increase surface wettability, addressing the low activity in the inherent apolar nature of various heterogeneous photocatalysts.^36^ Similarly, good dispersion can facilitate the easy processing of organic molecules. Third, the heterogeneous nature of extended aggregates can be used to recover and recycle the photocatalysts as well as to tune the interface for photocatalytic reactions.^37^ Fourth, supramolecular protocols for the formation of aggregates could allow relatively easy purification and eliminate batch-to-batch variations. Finally, organic “soft materials” benefit from properties such as relatively low toxicity, light weight, general affordability, and abundance on Earth.^38^

It should also be taken into consideration that the light absorption depends on the band energy gap (HOMO–LUMO for molecules and valence–conduction bands for semiconductors) and that the absolute bands positions determine the thermodynamic driving force for specific reactions. In the following sections we will also refer to co-catalysts and sacrificial agents. Indeed, in most cases it is necessary to add a dedicated co-catalyst (electrocatalyst) and a sacrificial agent that interact with photogenerated electrons and holes, making the light-absorbing unit essentially a photosensitiser. The addition of electrocatalysts enables certain photocatalytic reactions, such as H_2_ evolution, O_2_ production or CO_2_ reduction. In other cases, such as environmental remediation and H_2_O_2_ production, the addition of co-catalysts can be avoided since the direct interaction of the semiconductor with molecular O_2_ can occur. Finally, we also mention photocatalytic apparent quantum yields (AQYs), sometimes also called apparent quantum efficiencies, which are calculated as the ratio between the number of reacted electrons (*e.g.*, 2× number of H_2_ molecules produced) and the number of incident photons of a certain wavelength, in the system. Since photocatalysis can be affected by a variety of parameters, including light sources and illumination conditions, we focus on the AQY at certain wavelengths rather than the product evolution rate.

## Self-assembly of chromophores in photocatalytic nanostructures

**Fig. 3:**
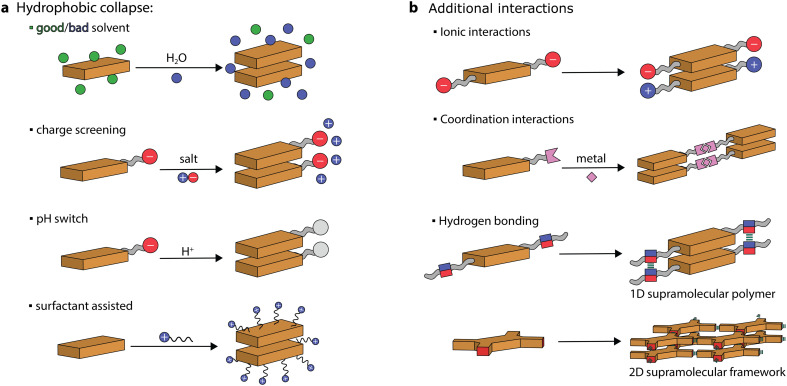
Chromophore aggregation triggered in aqueous media through various protocols. (a) Precipitation, charge screening, acid–base neutralization, and surfactant-assisted self-assembly. (b) Aggregation of dyes through hydrophobic collapse and π–π stacking aided through additional interactions, which include complementary charges, coordination interactions, and hydrogen bonding.

Chromophores, made of π-conjugated systems, usually possess low solubility in water and their tendency is to self-assemble into robust aggregates due to a combination of hydrophobic and π–π stacking interactions. Large hydrophobic surfaces tend to aggregate in aqueous media both through entropic (hydration water being released in the bulk) and enthalpic (decrease of interfacial area with water) driving forces.^20^ Therefore, to prepare self-assembled nanostructures in aqueous media, the chromophore monomers (or discrete aggregates) need to be first solubilized (or dispersed) and then their aggregation can be triggered. This can be achieved through various protocols. For instance, supramolecular artificial antennas have been prepared using different procedures, including precipitation from good to bad solvents, ionic self-assembly, acid–base neutralization, and surfactant-assisted self-assembly (see Fig. 3). These protocols, that have been exploited to prepare other systems as well, will be presented and discussed in the following sections.

### Precipitation

The main approach is to dissolve the chromophore in a good solvent and then to induce its self-assembly by addition of a bad solvent. This is usually achieved by injecting an organic solution of the dye into water, which induces the hydrophobic collapse of monomers into supramolecular structures.^39–41^

Organic molecules comprised of electron-rich donor and electron-deficient acceptor units can facilitate charge transfer in the excited state,^42,43^ ultimately improving exciton dissociation and enhancing visible light absorption. One of the most exploited self-assembled dye system in photocatalysis is based on perylene diimides (PDIs, perylene-3,4,9,10-tetracarboxylic diimides). PDIs are characterized by a strong conjugation between the electron-rich perylene core and the electron-poor imide groups, resulting in an exceptional acceptor–donor–acceptor scaffold. Unsubstituted PDIs can be initially dissolved in concentrated sulfuric acid, and then its hydrophobic collapse can be induced by the addition of water.^44^ Crystalline materials can be obtained due to the strong π–π stacking of H-aggregated molecules aided by lateral hydrogen bonding (–N–H⋯O=). Strong π–π stacking also results in a high orbital overlap between PDI molecules, leading to a deeper valence band of the self-assembled organic semiconductor (+2.20 eV *vs.* NHE) compared to the LUMO of the monomeric PDI (+1.61 eV *vs.* NHE), which was exploited for water oxidation to O_2_, upon visible light irradiation and in the presence of a sacrificial acceptor (Ag^+^).^44^ It was suggested that light irradiation leads to photogenerated electrons and holes that are delocalized along the π–π stacking direction.

**Fig. 4:**
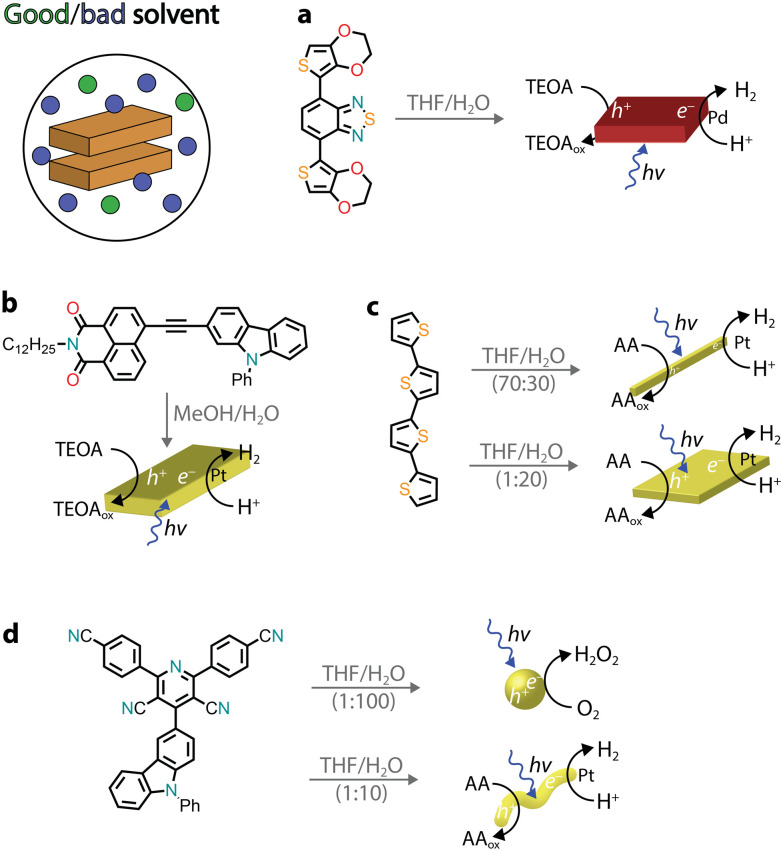
Representative examples of self-assembled nanostructures used in photocatalysis prepared by precipitation. Abbreviations: THF: tetrahydrofuran, TEOA: triethanolamine, and AA: ascorbic acid.

Several other chromophores, consisting of donor–acceptor motifs, have been reported to self-assemble into nanostructures through precipitation protocols and possess photocatalytic activity, ranging from H_2_ evolution^45^ to the photooxidation of organic substrates^46^ and environmental remediation.^47^ Recently, organic donor–acceptor–donor molecules based on thiophene–benzothiadiazole–thiophene (TBT) were precipitated (THF/H_2_O) and used for photocatalytic H_2_ evolution (Fig. 4a).^48,49^ In the presence of the triethanolamine (TEOA) sacrificial donor, the rod-like aggregates photoproduced H_2_ with an AQY of approximately 2% (400–450 nm), which was attributed to a combination of broad visible light absorption, a small band gap, wettability, and long exciton lifetimes. Furthermore, it was observed that no additional co-catalyst was added, and the residual palladium from cross-coupling was found to impact the photocatalytic activity.

The presence of donor–acceptor groups in monomer dyes can result in molecules with large dipole moments, facilitating the self-assembly process through dipole–dipole interactions. A naphthalene monoimide–phenyl carbazole monomer (NMI–Czl, Fig. 4b) was found to form nanoribbons upon precipitation, driven by alternate stacking of electron-rich moieties (carbazole, Czl) with electron-deficient groups (naphthalene monoimide, NMI).^50^ It was observed that long-lived charge-separated excitons were generated upon photoexcitation, which were responsible for the photocatalytic activity. Specifically, H_2_ evolution was observed in the presence of a platinum co-catalyst and TEOA as a sacrificial donor with an AQY of 1.3% at 400 nm.

By precipitating a chromophore from a good solvent to a bad solvent, the volume-to-volume ratio between these two solvents can play an important role in tuning the hydrophobicity and, consequently, the packing between monomers. It is perhaps not surprising that different packings, which usually translate into different morphologies, can lead to dissimilar photocatalytic activity.^51–54^ In one example, a quaterthiophene (QTH) molecule was observed to form crystalline nanorods in a THF/H_2_O mixture (7 : 3 v/v), whereas a nanosheet morphology was noted when a THF solution was injected into H_2_O (Fig. 4c).^54^ It was proposed that the monomers arrange either in a head-to-tail fashion, leading to nanorods, or in a slipped stacking arrangement, resulting in a nanosheet morphology. In the latter, enhanced photoexcited charge transfer was observed, leading to higher H_2_ photocatalytic activity (in the presence of Pt co-catalysts and the ascorbic acid sacrificial donor). Notably, for quaterthiophene nanosheets, it was claimed that a solar-to-hydrogen efficiency of 18% could be reached when a Pt co-catalyst and a 4-methylbenzyl alcohol sacrificial donor were used. Another recent example is a donor–acceptor organic molecule that was shown to exhibit two different aggregate states, depending on the volume ratio of THF/H_2_O used for precipitation (Fig. 4d).^55^ Due to the difference in packing, the two morphologies also showed different photocatalytic activities with the nanospheres producing H_2_O_2_ and the nanofibers promoting H_2_ production. It was proposed that the excitons underwent charge separation facilitated by the nanofiber crystalline structure, while the amorphous nanospheres showed better electron transfer to O_2_ (critical in the photocatalytic H_2_O_2_ production).

### Charge screening

Chromophores that carry charged headgroups can dissolve in aqueous solutions due to the electrostatic repulsion between the ionic groups on the monomers. However, upon the addition of salts, the electrostatic repulsion can be screened, which leads to the hydrophobic collapse of chromophores into ordered aggregates.

**Fig. 5:**
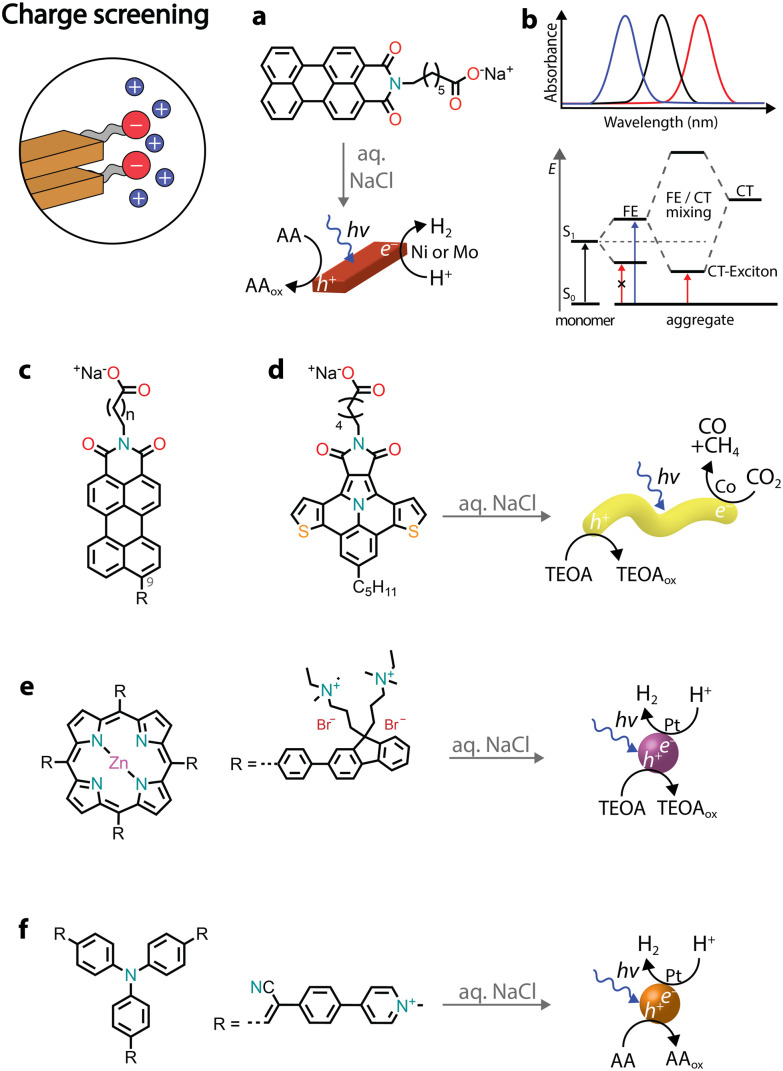
Illustrative examples in which charge screening was employed to self-assemble amphiphilic chromophores and used for the photoproduction of solar fuels.

The self-assembly of several perylene monoimides (PMIs), carrying a carboxylate alkyl chain at the imide position, has been thoroughly studied in the presence of salts. A decade ago, a pioneering study reported the formation of crystalline nanoribbons after the addition of salts to aqueous solutions of a PMI amphiphile (Fig. 5a).^56^ The addition of salt is responsible for screening the negatively charged headgroups of PMIs, causing hydrophobic collapse into ordered nanostructures. Another notable observation was that the formation of extended nanostructures, and their entanglement, led to the formation of hydrogels. It was also reported that hydrated nanostructures, in which the supramolecular photosensitizers and nickel DuBois catalysts were co-localized, led to better photocatalytic H_2_ evolution in the presence of sacrificial ascorbic acid compared to dried gels on solid substrates.

Recently, it was reported that enhancing the porous scaffold within the PMI hydrogel led to better photocatalytic properties.^57^ Thermal annealing (at 95 °C for 1 hour) of an aqueous PMI amphiphile solution containing salts led to the growth of large crystalline nanoribbons. The resulting crystalline polymers exhibited a more porous structure compared to control samples, leading to enhanced diffusion of [Mo_3_S_13_]^2−^ catalysts and the ascorbic acid sacrificial donor, ultimately contributing to a better photocatalytic H_2_ evolution.

PMI crystalline assemblies have interesting photophysical properties.^58^ As mentioned above, chromophore assemblies can form H- and J-aggregates that display blue- and red-shifted absorption relative to the monomer absorbance maxima, respectively. In addition to the long-range coulombic coupling of the chromophore's transition dipole moments (TDMs), in organic crystals also short-range charge transfer (CT) coupling between π-orbitals can affect the formation of excitons. While TDM coupling is primarily influenced by the orientation of chromophores in space, CT coupling is very sensitive to sub-Å molecular displacements. When crystalline PMI assemblies are formed, the orbital orientation between pairs of molecules becomes identical across the crystal, resulting in pronounced CT coupling. The two modes of coupling can even mix, giving rise to the creation of a completely new electronic structure known as the CT exciton (Fig. 5b). Therefore, in PMI assemblies, in addition to the typical absorption blue-shifted peak of H-aggregates, a new red-shifted absorbance feature is observed due to the mixing of TDM and CT states.

Several chemical modifications were reported on PMI amphiphiles, both on the charged headgroup and the perylene core (Fig. 5c). For example, increasing the distance between the charged headgroup and the aromatic core resulted in aggregates with higher orbital overlap and more exciton splitting, leading to better photocatalytic H_2_ evolution.^59^ Additionally, different substituents were introduced at position 9 of the perylene core, influencing the dipole moments and energy levels of the chromophores.^60,61^ Electron-poor substituents (such as –CN) led to nanostructures with no photocatalytic activity, due to the decreased dipole–dipole interactions between monomers and the lowered conduction band below the catalyst's reduction potential. Electron-rich substituents (such as –NH_2_ or –OCH_3_) gave nanostructures with photocatalytic H_2_ activity, but lower when compared to the unsubstituted PMI amphiphiles. Increased dipole–dipole interactions were found to result in a different crystalline packing with lower exciton splitting.^60,62^ Introducing substituents with varying steric demands was found to influence crystal packing and, subsequently, the formation of CT excitons.^63,64^ Less sterically demanding substituents led to molecules that quickly crystallized in water, but with no CT exciton formation and a low H_2_ photocatalytic activity. Bulkier substituents needed addition of salt and thermal annealing to crystallize, but resulted in crystalline nanoribbons with red-shifted CT excitons that could efficiently photosensitize the [Mo_3_S_13_]^2−^ catalyst for H_2_ evolution in the presence of ascorbic acid.

Due to the LUMO energy of PMIs, it is expected that the corresponding nanostructures could predominantly photosensitize proton reduction catalysts. By substituting the perylene core with more electron-rich ones, such as diareno-fused ullazines, supramolecular polymers capable of sensitizing CO_2_ reduction electrocatalysts could be obtained (Fig. 5d).^65^ Ullazine imides were found to drive the reduction of CO_2_ to CO and CH_4_ in the presence of dicobalt catalysts and sacrificial TEOA over longer periods of time when compared to homogeneous photosensitizers.

The chromore's photophysical properties, other than CT formation, could also be affected by the addition of salts, ultimately contributing to improved H_2_ evolution. In one example, an octacationic zinc porphyrin (Zn tetraphenylporphyrin fluorene derivative, ZnTPP-FN) was charge screened in the presence of sodium chloride, leading to the formation of aggregated microspheres (Fig. 5e).^66^ While no long-range order was observed, the aggregation resulted in structures with enhanced communication with the Pt catalysts and faster transfer of photogenerated holes to the TEOA donor. The charge screening by various halide salts was also found to influence the photocatalytic nanostructures (Fig. 5f).^67^ Specifically, addition of iodide salts was found to increase intersystem crossing, resulting in triplet states with longer lifetimes and achieving an AQY of almost 10% at 500 nm in the presence of Pt co-catalysts and sacrificial ascorbic acid.

### pH switch

Similarly to charge screening, acid–base neutralization of charged chromophore amphiphiles also leads to their hydrophobic collapse into nanostructures. Most commonly, chromophores substituted with carboxylic acids are dissolved in water by the addition of a base (such as NaOH), which is followed by the addition of an acid to induce self-assembly through hydrophobic collapse.

**Fig. 6:**
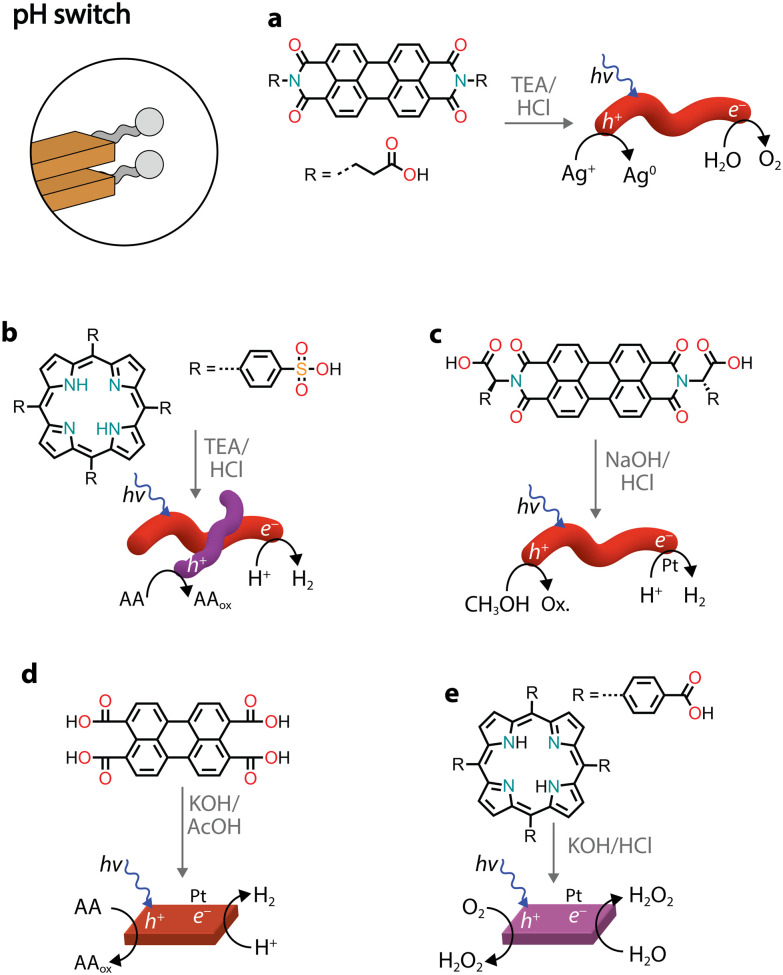
Representative examples of supramolecular nanostructures obtained through acid–base neutralization. Abbreviations: TEA: triethylamine and AcOH: acetic acid.

Several perylene diimide (PDI) derivatives have been well studied as model chromophore amphiphiles for the formation of photocatalytic nanostructures through acid–base neutralization (Fig. 6a).^68,69^ PDIs can be synthesized in high yields through condensation reactions between perylene dianhydride and substituted amines. A PDI substituted with propanoic acid, in particular, has been exploited for its photocatalytic properties in O_2_ evolution,^70^^1^O_2_ generation,^71^ and even in environmental remediation.^72,73^ In one example, the relatively deep valence band (+1.52 V *vs.* NHE) of the PDI nanofibers was exploited for the water oxidation reaction (+1.23 V *vs.* NHE).^70^ In the presence of Ag^+^ sacrificial acceptors, it was observed that the O_2_ quantum yield could reach 0.5% at 600 nm. Also in this case, the catalytic activity could be attributed to the formation of π–π stacks that allow separation and migration of the photogenerated carriers. It is interesting to note that the photogenerated electron–hole separation and migration could be tuned by modifying the linker length between the PDI core and the carboxylic acid^71^ or by self-sorting with nanostructures made of other chromophore amphiphiles (Fig. 6b).^74^

Conjugates of natural amino acids and PDIs have been carefully studied for their self-assembly into nanofibers (Fig. 6c).^75–77^ The carboxylic acid group on the amino acid side groups allow the chromophores to be solubilized at high pH. The addition of a dilute acid then reduces solubility through protonation of the side groups, initiating further self-assembly into one-dimensional nanostructures. In addition to pH, the aggregation state can also be controlled by the addition of different amounts of methanol, which serves as a sacrificial electron donor in photocatalytic H_2_ evolution with Pt co-catalysts.^77^ It was also noted that the amino acid side chain also played an important role in the local packing within the aggregates, resulting either in strong or weak mixing of Frenkel and CT excitons.

Recently, perylene tetracarboxylic acid (PTCA) was first dissolved in water through hydrolysis of the anhydride precursor and, upon the addition of acetic acid, self-assembled nanosheets were formed (Fig. 6d).^78^ The nanostructures in the presence of Pt co-catalysts and sacrificial ascorbic acid showed H_2_ evolution up to AQY = 13.5% at 420 nm. The activity was attributed to a combination of exciton coupling between π–π stacked molecules and hydrophilic carboxylic acids on the surface that come into direct contact with protons used for H_2_ production.

Porphyrin nanosheets, prepared through acid–base neutralization, have been reported for H_2_ or O_2_ evolution^79^ and very recently for H_2_O_2_ photoproduction as well (Fig. 6e).^80^ In the latter case, it was proposed that H_2_O_2_ could be produced through both reductive (O_2_ to H_2_O_2_) and oxidative (H_2_O to H_2_O_2_) pathways. The involvement of both photogenerated electrons and holes allowed the system to achieve a quantum efficiency of 14.9% at 420 nm.^80^ While the reductive H_2_O_2_ production occurred as expected through O_2_ reduction to superoxide radicals, which subsequently react with protons to form H_2_O_2_, the oxidative process did not necessitate a sacrificial donor and managed to use the holes in water oxidation to H_2_O_2_. It was proposed that the carboxylic groups on the chromophores could be oxidized to peroxy acid groups, which in the presence of water could thermally decompose into H_2_O_2_. This study paves the way forward for the use of organic nanostructures in solar fuel production without the need for additional co-catalysts and sacrificial donors.

### Surfactant-assisted self-assembly

Among various protocols, surfactant-assisted self-assembly has been particularly studied in the formation of porphyrin nanostructures.^81^ In particular, using surfactants allows the use of hydrophobic chromophores that have not been chemically modified. It permits good morphological tuning, stabilizes nanostructures in water, enables controlled growth of nanostructures, and is compatible with the protocols already discussed.

**Fig. 7:**
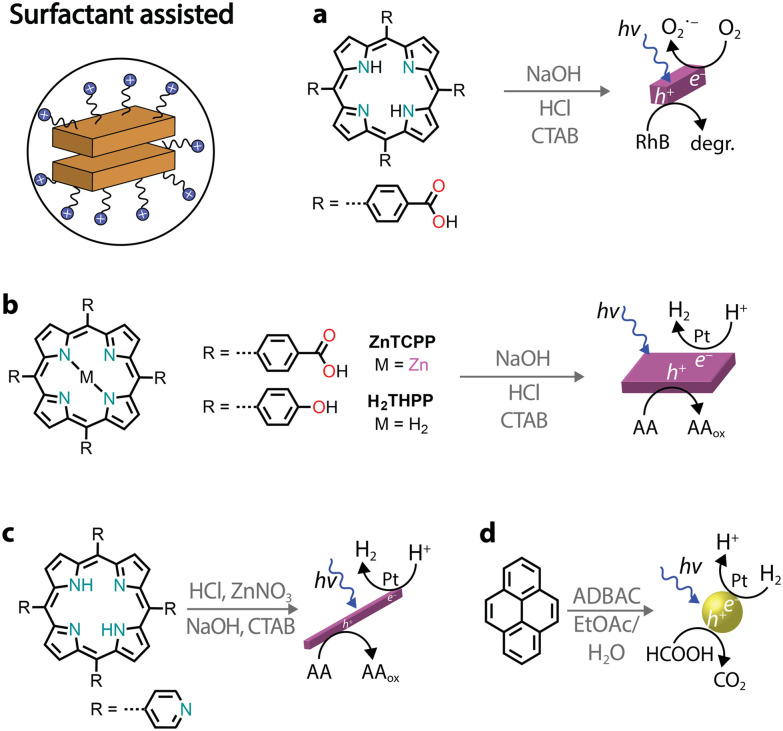
Illustrative examples of photocatalytic nanostructures obtained through surfactant-assisted self-assembly.

A zinc tetra(4-pyridyl)porphyrin, first solubilized in an acidic solution and then injected into a basic solution, yields amorphous irregular particles.^82^ However, in the presence of surfactants, porphyrin nanocrystals can be obtained (Fig. 7a).^82–84^ Furthermore, it was observed that nanocrystal nucleation and growth are under kinetic control, which was used to obtain different morphologies. Similarly, a deprotonation–protonation reaction in a porphyrin carrying acidic groups can lead to the formation of nanocrystalline aggregates.^85–88^ For example, 5,10,15,20-tetrakis(4-(hydroxyl)phenyl)porphyrin (THPP) in the presence of a surfactant (cetyltrimethylammonium bromide, CTAB) yielded aggregates confined within surfactant micelles (Fig. 7b).^86^ The formation of crystalline nanostructures exhibited higher photocatalytic H_2_ evolution with a Pt co-catalyst and sacrificial ascorbic acid, especially when compared to the original porphyrin powders.

As an alternative to the acid–base neutralization of substituted porphyrins, surfactants can also be used to aid precipitation^89,90^ or facilitate the formation of microemulsions.^91^ In the latter, oil-in-water emulsions can be heated to evaporate the organic solvent, triggering the self-assembly of nanostructures within the surfactant droplets (Fig. 7c).^92,93^ Recently, it was also shown that chromophores other than porphyrins can also be benefit from aggregation through the surfactant microemulsion procedure.^94^ For example, the photocatalytic H_2_ activity of pyrene aggregates can reach an AQY of 20% at 400 nm in the presence of a platinum co-catalyst and sacrificial formic acid (Fig. 7d).

### Hydrogen bonding-aided self-assembly

While π–π interactions can drive the formation of chromophore stacks in aqueous solutions, by introducing lateral groups that are able to hydrogen bond,^95^ monomers can be guided to form supramolecular nanostructures with improved exciton coupling between the dyes.

**Fig. 8:**
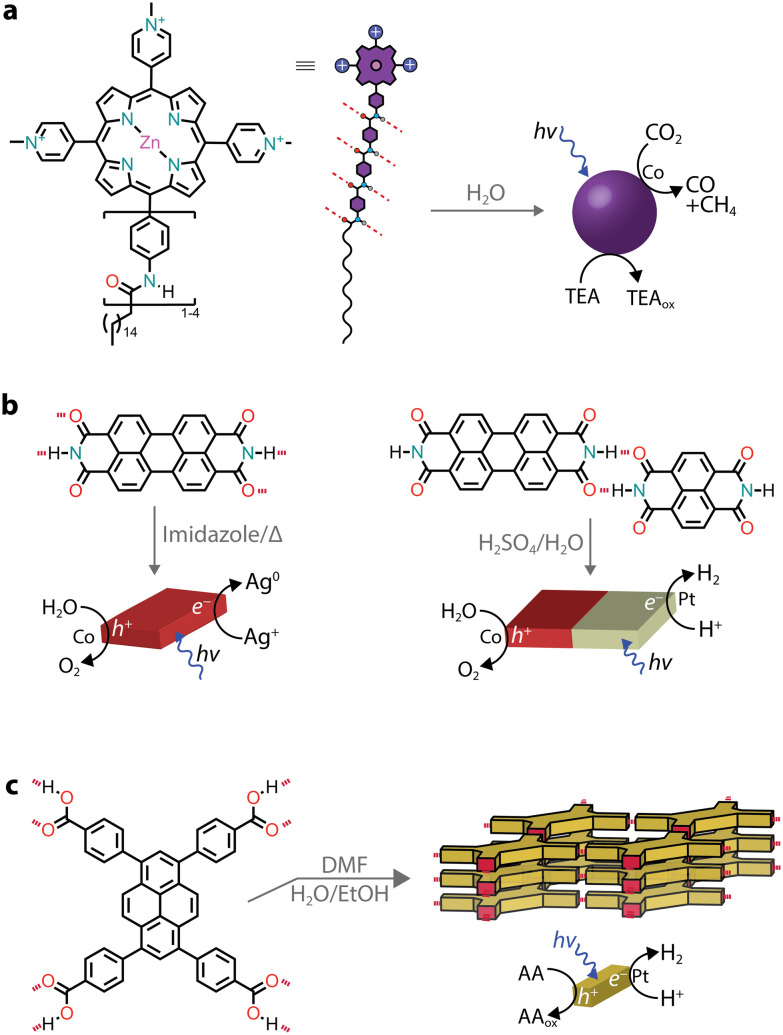
Hydrogen bonding can be used to aid the supramolecular organization of chromophores.

For instance, a tricationic zinc porphyrin was modified with aramid linkers, which guided the self-assembly of the chromophore amphiphiles into micelles (Fig. 8a).^96,97^ The quadruple hydrogen bonding between monomers led to photostable nanostructures, while the solubilized monomer was found to photobleach quickly.^96^ When a negatively charged cobalt porphyrin catalyst was added to interact with a positively charged photosensitizer, the hierarchical nanostructure created a micro-environment capable of driving CO_2_ reduction to H_2_, CO, and CH_4_ with an initial AQY of 15% (at 450 nm) in the presence of a triethylamine sacrificial donor.^96^ Similar to the aramid linker, chromophores with side chains capable of hydrogen bonding (such as peptide nucleic acids and peptides) were reported to lead to nanostructures with photosensitizing capabilities.^98–100^

Several PDI derivatives were reported to form lateral hydrogen bonding between the dye stacks, resulting in nanosheets or nanoribbon morphologies.^44,68–70,101–103^ While most of them were discussed in previous sections, since excitonic coupling occurs along the π–π stacking directions, it has recently been shown that lateral hydrogen bonds can be exploited for the formation of heterojunctions (Fig. 8b).^104^ The hydrogen bonding between two organic self-assembled nanostructures was reported to create a Z-scheme heterostructure for overall water splitting.^104^ The photogenerated traps on PDI nanocrystals could catalyse the production of O_2_ together with a cobalt co-catalyst, while the electrons on naphthalene diimides lead to the production of H_2_ aided by a platinum co-catalyst. The AQY for O_2_ production reached 5.2% (at 380 nm) in the presence of an Ag^+^ sacrificial acceptor.

An interesting case is when discrete tectons are pre-designed to self-assemble with intermolecular hydrogen bonds to obtain crystalline and porous materials, known as hydrogen-bonded organic frameworks (HOF, Fig. 8c). When organic chromophores are devised to carry multiple highly directional complementary H-bonding interactions, frameworks can be obtained. These frameworks take advantage of the π–π stacking between the layers for excitonic coupling and the porous nature of the scaffold.^105–108^ Recently, a HOF based on 1,3,6,8-tetrakis(*p*-benzoic acid)pyrene (TBAPy) was reported to reach an AQY of 28.6% (at 420 nm) in sacrificial photocatalytic H_2_ evolution (ascorbic acid and Pt co-catalysts).^109^ This high activity was attributed to the fast transfer of photogenerated excitons to the adjacent hydrophilic micropores, which reduces the loss of excitons through recombination during their migration. Previously, it was also shown that the same crystalline framework had a 200-fold higher photocatalytic activity compared to the amorphous material.^110^ It was further reported that by changing the linker length between the pyrene core and the carboxylic acids, two main effects can be observed: (i) the pores are enlarged, aiding the diffusion of molecules within the frameworks^111,112^ and (ii) the additional π–π stacking between linkers can greatly enhance the chemical and thermal stability of the HOFs.^111^ In addition to H_2_ photoproduction, pyrene-based HOFs were also reported for photooxidation of sulfides to sulfoxides through singlet oxygen production.^111,113^

### Interaction with catalysts, (macro)molecules and materials

For improved photocatalytic activities, it is crucial to interface the self-assembled chromophores with metals, catalysts, and materials. Alternatively, the self-assembly of dyes can be assisted, and even templated, by addition of other molecules or polymers.

**Fig. 9:**
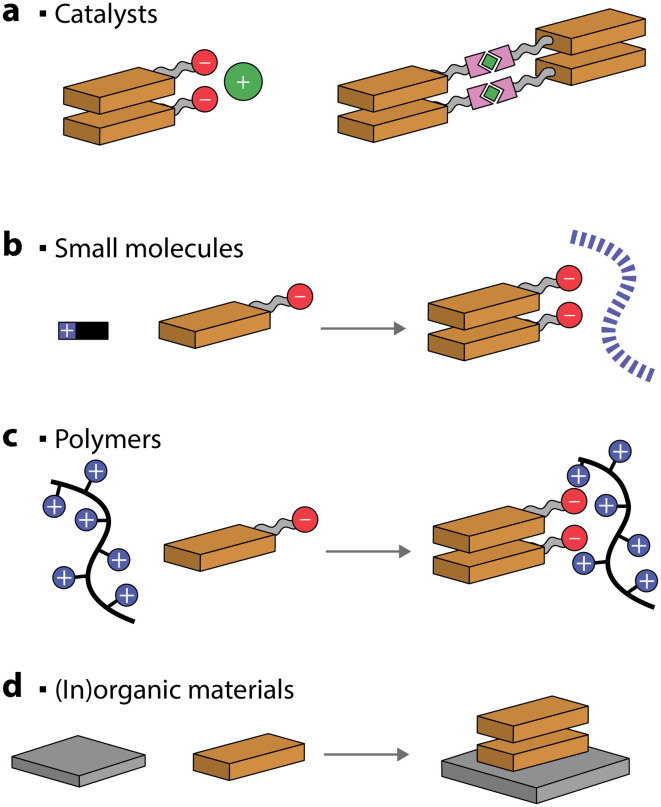
Schematic representation of self-assembled chromophores that can be co-assembled with co-catalysts, small molecules, polymers, or (in)organic materials.

Ionic interactions between complementary charges are commonly used to drive interactions between charged photosensitizers and co-catalysts (Fig. 9a).^114–116^ It has been suggested that placing complementary positive or negative charges either on the nanostructures or the catalysts is not interchangeable and can lead to a difference of up to 5 times in the photocatalytic production of H_2_.^117^

An interesting approach to creating self-assembled chromophore photocatalytic nanostructures is to take advantage of coordination bonds. Metal–ligand coordination can be exploited to decrease the solubility of the dye in the solvent system and thus drive the organized aggregation through π–π interactions.^118,119^ Even more stimulating is the case where the metals added also serve as catalysts for various chemical reactions (Fig. 9a).^120,121^ For example, a coordination polymer gel, made through ruthenium–polypyridyl complexes, was utilized to drive CO_2_ to CH_4_ photoreduction with an AQY of 7.7% (at 400 nm) in the presence of triethylamine and 1-benzyl-1,4-dihydronicotinamide sacrificial donor. Similarly, a PDI–zirconium self-assembled nanostructure showed good photocatalytic H_2_ ability, reaching 11.7% AQY (at 630 nm), in the presence of sacrificial ascorbic acid.^122^ Another strategy used to enhance the photocatalytic performance is anchoring co-catalysts to self-assembled nanostructures through metal–ligand interactions.^122–125^

Oppositely charged small molecules and chromophores can spontaneously self-assemble into hierarchical nanostructures in the presence of salts and acids.^126–128^ Small molecules, such as peptides, can typically form supramolecular polymers by themselves. However, when chromophores are added, a long-range order of the latter can be obtained (Fig. 9b). This can result in the excitons being delocalized over multiple porphyrin molecules, which can be utilized for photocatalytic H_2_ evolution in the presence of Pt catalysts and sacrificial ascorbic acid.^126–128^ Similarly, hierarchical self-assembled nanostructures were obtained through ionic interactions between Fmoc-dipeptides and tin porphyrins.^129^ The resulting nanostructures were found to photooxidize H_2_O to O_2_ in the presence of iridium oxide as a co-catalyst and persulfate as a sacrificial electron acceptor.

Charged covalent polymers were also shown to act as templates for inducing the self-assembly of chromophores (Fig. 9c).^130^ For example, PMI amphiphiles were shown to self-assemble into crystalline nanoribbons when loaded into positively charged covalent hydrogels.^130^ It was observed that the presence of a charged polymer had a similar effect on the chromophore self-assembly to charge screening with salts. Also in this case, the photocatalytic H_2_ evolution, in the presence of a thiomolybdate catalyst and sacrificial ascorbic acid, was observed. In another example, a positively charged porphyrin was self-assembled on anionic poly(styrene sulfonate) polymeric brushes.^131^ The polymer, acting as a nanoscale support for controlled porphyrin aggregation, was utilized for light-induced iodide oxidation.

Complementary charges were also employed to self-assemble positively- and negatively-charged chromophores, constructing photosensitizing nanostructures for H_2_ evolution (in the presence of Pt and TEOA),^132,133^ to form donor–acceptor nanostructures that promote charge separation^134^ and could potentially be used for nanostructures interaction with electron mediators in photocatalytic applications.^135^

Preparation of organic–inorganic composites is another common strategy to improve the photocatalytic activity, mainly due to the improved separation efficiency of photogenerated electron–hole pairs (Fig. 9d). One of the most common self-assembled organic–inorganic materials is PDI/TiO_2_.^136–138^ It was proposed that the two can interact through hydrogen bonding and it was noted that the hybrid could reach an AQY for H_2_ production of 70% (at 365 nm) in the presence of Pt as a co-catalyst and methanol as a sacrificial donor.^139^ TiO_2_ could also be deposited *in situ* on one-dimensional PDI nanofibers and the composite was utilized for H_2_ evolution in the presence of a Pt co-catalyst and an amine sacrificial donor.^140,141^ Heterojunctions of PDIs have also been reported with Ag_2_S quantum dots,^142^ SnO_2_ quantum dots,^143^ Pd quantum dots,^144^ indium tin oxide,^145^ Bi_2_WO_6_,^146^ and Ti_3_C_2_T_*x*_ MXenes,^147^ to name a few. Another possibility for preparing heterojunction photocatalysts is to self-assemble PDIs in the presence of organic materials, such as graphene^148^ and carbon nitride.^149^ Other commonly utilized self-assembled chromophores to form composited are porphyrins, which have been reported in conjunction with, for example, TiO_2_ ^150–154^ and graphene.^155,156^ The common observation for all the mentioned composited is that their photocatalytic activity was higher when compared to the individual components, demonstrating that this is a viable strategy for further improving the efficiency of self-assembled chromophores.

### Energy landscapes of supramolecular photocatalysts

Depending on the strength and dynamics of non-covalent bonds, there can be a subtle interplay between kinetics and thermodynamics during aggregation. Therefore, a supramolecular aggregate might follow the kinetic pathway rather than the thermodynamic one. Experimental studies have shown that the outcome between kinetic and thermodynamic aggregates depends on the preparation methods, which span from temperature variation to solvent processing.^157^ In the context of supramolecular chromophore nanostructures, the kinetic and thermodynamic aggregates might possess different photocatalytic properties, especially if the excitonic coupling in the two states is different.

**Fig. 10:**
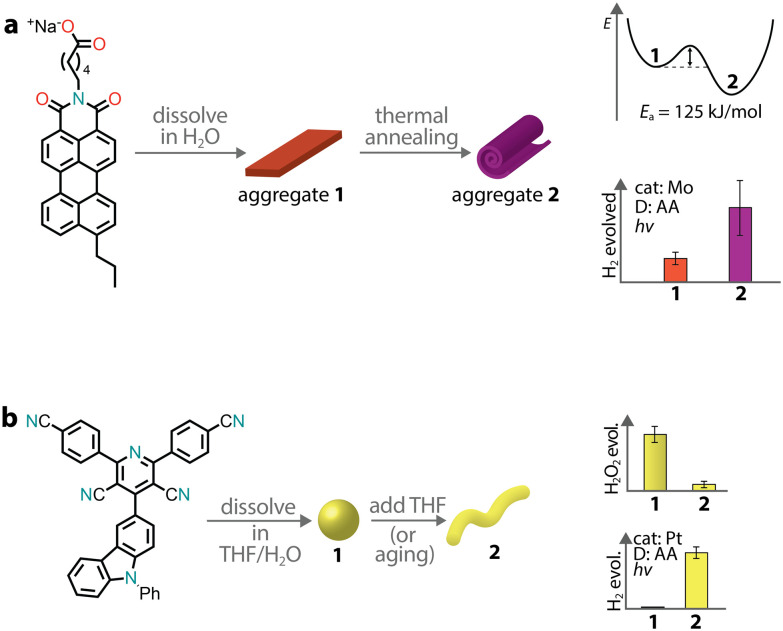
Aggregation leading to the formation of kinetic and thermodynamic products, which can have different photocatalytic activities.

This was the case for a PMI amphiphile with a propyl tail at position 9, which was observed to form two distinct supramolecular architectures with different photocatalytic activities (Fig. 10a).^158^ When the chromophore was dissolved in an aqueous salt solution, it formed crystalline ribbons. Upon thermal annealing, the ribbons were transformed into crystalline scroll-like nanostructures. Importantly, the transformation led to a three-fold improvement in photocatalytic H_2_ production with a thiomolybdate catalyst and ascorbic acid as a sacrificial donor. Only in the stable phase, the formation of CT excitons was observed, which were responsible for the enhancement of H_2_ evolution.

We already mentioned a donor–acceptor organic molecule that exhibited the formation of two different aggregate states in aqueous dispersion, depending on the THF/water ratio used in the precipitation procedure.^55^ It was also observed that amorphous nanospheres could transform into crystalline nanofibers upon aging, suggesting that the former are the kinetic product while the latter the thermodynamic one (Fig. 10b). This case is quite peculiar as one morphology (amorphous nanospheres) promoted H_2_O_2_ photoproduction, while the other (crystalline nanofibers) supported sacrificial H_2_ evolution.

In addition to aging and thermal annealing, there is also a need to develop alternative strategies to obtain thermodynamic aggregates. Since rapidly injecting a solution into a bad solvent could lead to the formation of kinetic aggregates, an *in situ* enzymatic hydrolysis method for self-assembled chromophores was proposed.^159^ Diketopyrrolopyrrole, functionalized with amino acid methyl esters, was found to produce supramolecular hydrogels after enzymatic hydrolysis. The hydrogels were used to produce ^1^O_2_, which was exploited for the oxidation of sulfides to sulfoxides.

## Conclusions

In this contribution, we have discussed the use of self-assembled chromophores in the field of photocatalytic energy conversion, with the representative examples summarized in Table 1. Due to their large π-conjugated structures, dye–dye stacking is driven by π–π and hydrophobic interactions, enabling the formation of aggregates. A particularly interesting case of chromophore amphiphile self-assembly is the formation of hydrogels with their three-dimensional porous networks allowing co-localization and easy diffusion of the species involved in catalysis.

**Table 1:** Overview of the representative examples of self-assembled nanostructures in photocatalytic applications

Compound^a^	Prep.^b^	Morphology	Gas evolution rate^c^/mmol g^−1^ h^−1^	TON^c^/mol_prod_ mol_cat_^−1^)	Light source	AQY^d^ (nm)/%	Catalyst^e^	Cond.^f^	Ref.
D–A molecule	THF/H_2_O	Nano/micro-rods	H_2_: 0.142	n.r.	Monochromatic LED (*λ* ≥ 420 nm)	2.0 (400–450 nm)	Pd (synthesis)	TEOA in H_2_O	48
NMI–Czl	MeOH/H_2_O	Nanoribbons	H_2_: 0.417 (3 h)	n.r.	Xe lamp (*λ* ≥ 400 nm)	1.3 (400 nm)	Pt (photodep.)	TEOA in H_2_O	50
QTH	THF/H_2_O	Nanosheets	H_2_: 1.21 (12 h)	n.r.	Xe lamp (110 mW cm^−2^)	3 (365 nm)	Pt (photodep.)	MBA in H_2_O	54
D–A molecule	THF/H_2_O	Nanospheres	H_2_O_2_: 3.20 (1 h)	n.r.	Solar simulator (AM1.5G)	n.r.	—	H_2_O (O_2_ atm)	55
	THF/H_2_O	Nanofibers	H_2_: 31.85 (1 h)	n.r.		4.43 (420 nm)	Pt (photodep.)	AA in H_2_O	
PMI	NaCl aq.	Nanoribbons	H_2_: 0.63 (18 h)	3.4 × 10^2^ (18 h)	Halogen bulb (250 W cm^−2^)	n.r.	Ni DuBois (molecular)	AA in H_2_O	56
PMI	NaCl aq.	Nanoribbons	n.r.	7.5 × 10^3^ (110 h)	Halogen bulb (250 W cm^−2^)	0.06 (505 nm)	[Mo_3_S_13_]^2−^ (molecular)	AA in H_2_O	57
	NaCl aq. (thermal annealing)		n.r.	1.35 × 10^4^ (110 h)		0.13 (505 nm)			
PMI	NaCl aq. (thermal annealing)	Nanoribbons	n.r.	H_2_: 1.5 × 10^3^ (18 h)	Halogen bulb (250 W cm^−2^)	n.r.	[Mo_3_S_13_]^2−^ (molecular)	AA in H_2_O	63
PMI	NaCl aq. (thermal annealing)	Nanoribbons (helical)	n.r.	H_2_: 1.1 × 10^4^ (18 h)	Halogen bulb (250 W cm^−2^)	n.r.	[Mo_3_S_13_]^2−^ (molecular)	AA in H_2_O	158
Ullazine imides	NaCl aq. (thermal annealing)	Nanofibers	n.r.	CO: 4.6 × 10^4^	450 nm LED (200 mW cm^−2^)	n.r.	Dicobalt criptate (molecular)	TEOA in CH_3_CN/H_2_O (CO_2_ atm)	65
				CH_4_: 1.5 × 10^4^					
				H_2_: 26 (144 h)					
ZnTPP-FN	NaCl aq.	Microspheres	H_2_: 10.8 (6 h)	n.r.	Xe lamp (AM1.5G, 100 mW cm^−2^)	n.r.	Pt (photodep.)	TEOA in H_2_O	66
D–A molecule	NaI aq.	Nanoparticles	H_2_: 460 (*ca.* 2.5 h)	*Ca*. 9 × 10^3^ (12 h)	Xe lamp (3.3 mW cm^−2^, *λ* ≥ 450 nm)	9.8 (500 nm)	Pt (photodep.)	AA in H_2_O	67
PDI	H_2_SO_4_/H_2_O	Nanosheets	H_2_: 1.1 (*ca.* 1.5 h)	n.r.	Xe lamp (385 mW cm^−2^, *λ* ≥ 420 nm)	n.r.	Pt (photodep.)	AA in H_2_O	68
PDI	TEA/HCl and H_2_O	Nanofibers	O_2_: *ca*. 0.02	n.r.	Xe lamp (48 mW cm^−2^, 600 nm)	0.56 (600 nm)	—	AgNO_3_ in H_2_O	70
PDI/TPPS	TEA/HCl and H_2_O	Nanofibers	H_2_: 30.36 (3 h)	n.r.	Xe lamp (600 mW cm^−2^)	3.81 (650 nm)	Pt (photodep.)	AA in H_2_O	74
PDI	NaOH/HCl and H_2_O	Nanofibers	H_2_: 0.003 (4 h)	1.58 × 10^2^ (307 h)	Xe lamp (100 mW cm^−2^)	0.018 (365 nm)	Pt (NPs)	MeOH in H_2_O	75
PTCA	KOH/CH_3_COOH and H_2_O	Nanosheets	H_2_: 41.8 (2.5 h)	n.r.	Xe lamp (100 mW cm^−2^, *λ* ≥ 420 nm)	13.5 (420 nm)	Pt (photodep.)	AA in H_2_O	78
TCPP	KOH/HCl and H_2_O	Nanosheets	H_2_: 0.041 (6 h)	n.r.	Xe lamp (152 mW cm^−2^)	n.r.	—	TEOA in H_2_O	79
			O_2_: 0.036 (6 h)					AgNO_3_ in H_2_O	
TCPP	KOH/HCl and H_2_O	Nanosheets	H_2_O_2_: 1.25 (4 h)	n.r.	Xe lamp (*λ* ≥ 420 nm)	14.9 (420 nm)	—	H_2_O (O_2_ atm)	80
ZnTPyP	HCl/NaOH and CTAB H_2_O	Nanocrystals	H_2_: 47.1 (5 h)	n.r.	Xe lamp (*λ* ≥ 420 nm)	n.r.	Pt (photodep.)	AA in H_2_O	83
THPP	NaOH/HCl and CTAB H_2_O	Nanorods	H_2_: 19.5 (5 h)	n.r.	Xe lamp (90 mW cm^−2^, *λ* ≥ 420 nm)	n.r.	Pt (photodep.)	AA in H_2_O	86
ZnTCPP/THPP	NaOH/HCl and CTAB H_2_O	Nanosheets	H_2_: 41.4 (9 h)	n.r.	Xe lamp (632 mW cm^−2^)	2.07 (420 nm)	Pt (photodep.)	AA in H_2_O	88
InTPP	CHCl_3_/H_2_O and CTAB	Nanorods	H_2_: 845.4 (5 h)	n.r.	Xe lamp (*λ* ≥ 400 nm)	n.r.	Pt (photodep.)	AA in H_2_O	93
Pyrene	EtOAc/ADBAC and H_2_O	Nanoparticles	H_2_: *ca*. 0.16 (4 h)	n.r.	LED lamp (50 W, 400 nm)	20.8 (400 nm)	Pt (photodep.)	Formic acid	94
Zn porphyrin aramid	H_2_O	Nanomicelles	CH_4_: 8.5, CO: 2.8, H_2_: 1.6 (30 d)	CH_4_: 6.6 × 10^3^, CO: 2.2 × 10^3^, H_2_: 1.2 × 10^3^ (30 d)	Xe lamp (AM1.5G, *λ* ≥ 420 nm)	15.1 (450 nm)	Co complex (molecular)	TEA in H_2_O (CO_2_ atm)	96
NDINH/PDINH	H_2_SO_4_/H_2_O–ethylene glycol	Nanowires	H_2_: 0.32	n.r.	Xe lamp (783 mW cm^−2^)	3.36 (Pt, TEOA, 380 nm)	—	H_2_O	104
			O_2_: 0.15 (4 h)			5.16 (Co, AgNO_3_, 380 nm)			
TBAPy	DMF/H_2_O–EtOH	Framework	H_2_: 358 (2.5 h)	n.r.	Xe lamp (500 mW cm^−2^)	28.6 (420 nm)	Pt (photodep.)	AA in H_2_O	109
TBAPy	DMF/CHCl_3_–acetone	Framework	H_2_: 3.1 (20 h)	n.r.	Xe lamp (*λ* ≥ 420 nm)	4.1 (420 nm)	Pt (photodep.)	AA in H_2_O	110

a For brevity, here it is reported the abbreviation of the compound used in the main text and, for the complete chemical structure, it is suggested either to check the figures or the references.

b Solvents and reagents (or their mixtures) used for the preparation of the nanostructures.

c Values as reported (or estimated by us from figures when not explicitly mentioned) from the references; TON stands for turnover number; n.r. stands for not reported.

d Apparent quantum yield also referred to as the quantum efficiency and photonic efficiency in some of the references; n.r. stands for not reported.

e Metals and type of co-catalyst reported for photocatalytic activity (synthesis stands for the residual content from synthesis; photodep. stands for photodeposition; NPs stands for nanoparticles).

f Conditions used for photocatalytic testing which include sacrificial agents and solvents; TEOA is triethanolamine, AA is ascorbic acid, MBA is 4-methyl benzyl alcohol, and TEA is triethylamine.

The overlap of monomer molecular orbitals in the aggregate is beneficial for the formation of band-like electronic energy structures. Upon irradiation with visible light, the electrons in the valence band of the aggregates transition to the conduction band, generating electron–hole pairs. These pairs then undergo separation into free charges, ultimately driving redox reactions. Most commonly, these reactions lead to the production of H_2_, O_2_ evolution, and photodegradation reactions. However, examples of photooxidation of organic substrates, H_2_O_2_ production, and CO_2_ reduction have also been reported lately.

Despite the progress in this emerging field, several challenges lie ahead. While synthetic feasibility allows rational incorporation of functional groups, their effect on self-assembly, packing, and photocatalysis is not immediate and often is difficult to predict. Further efforts should also be directed toward the structural characterization of the aggregates. While the intrinsic nature of chromophores and their aggregates allows them to be studied by photophysical means (especially when coupled with theoretical calculations), the packing mode between the molecules is often not fully elucidated. In this context, having highly ordered assemblies can certainly be of help, but often this comes at the expense of the dynamic and reversible nature typical of supramolecular systems. For further advancements, it is also important to study the supramolecular polymerization processes, especially regarding the formation of kinetic and thermodynamic aggregates.

The photocatalytic activity of self-assembled chromophores also faces several challenges. Among these, stability against photodegradation, recyclability, photocatalytic mechanisms, and apparent quantum yields for a photocatalytic reaction are not always reported. A key question to be addressed in future research is whether self-assembled organic semiconductors can carry out the catalytic conversion by themselves or with the assistance of Earth-abundant, low-cost metal co-catalysts. While there are some indications that these systems can be integrated into electrodes, it is yet to be determined if photoelectrochemical systems can eliminate the need for sacrificial agents or enable new redox strategies. Expanding the portfolio of accessible chemical reactions, including organic photoredox transformations and photobiocatalysis, is another open question for the field. To further optimize the photocatalytic activity, attention should be placed on tuning the band-like structure of these materials, in which robotic experimentation could be of great help.^160,161^ The band structure could be tuned through chromophore co-assembling and alloying between crystalline aggregates,^162,163^ as well as attaining precise integration of the catalytic components (already demonstrated with covalent polymers).^164^ Improving the mechanical (and photophysical) properties of self-assembled chromophores, which could be achieved with hybrid supramolecular covalent materials,^165–167^ could allow the preparation of robust photocatalysts that might be even 3D printed.^168^ Finally, the dynamic nature of the aggregates could be used to obtain photocatalytic activity in an out-of-equilibrium state.^169,170^

Towards the full exploitation of self-assembled dyes for photocatalysis, it is desirable to address these open challenges. However, organic self-assembled materials have already provided a unique platform in photocatalysis. Promising research has shown the virtually infinite possibilities to tune these soft supramolecular photocatalysts for solar fuel generation.

## Conflicts of interest

There are no conflicts to declare.

## Supplementary Material
